# How to perform indexing of extravascular lung water data

**DOI:** 10.1186/cc10853

**Published:** 2012-03-20

**Authors:** S Wolf, A Riess, J Landscheidt, C Lumenta, L Schuerer, P Friederich

**Affiliations:** 1 Charite Berlin, Germany; 2 Klinikum Bogenhausen, Munich, Germany

## Introduction

Extravascular lung water (EVLW) is a marker for the severity of acute lung injury. To allow assessment of normal and pathologic states, traditionally EVLW data are either indexed to real or predicted body weight. Surprisingly and despite widespread use, this has so far not been validated in a larger cohort of subjects without cardiopulmonary compromise. The aim of the study was to prospectively evaluate a different ways of indexing EVLW data.

## Methods

EVLW was measured using single indicator transpulmonary thermodilution at predefined time points in 101 patients requiring elective brain tumor surgery. This database was used to investigate the properties of indexing EVLW data to real and predicted body weight, body surface area and body height.

## Results

EVLW indexed to predicted body weight was inversely correlated with a patient's body height (*P *< 0.001), while values indexed to real body weight remained inversely dependent on weight (*P *< 0.001). Indexing to estimated body surface area, again based on real or predicted body weight, provided no advantage. In contrast, indexing to body height presents an alternative method without dependence on physical properties or gender of a patient, yielding a uniform 95% confidence interval of normal values from 0.22 to 0.43 l/m.

## Conclusion

Traditional ways of indexing EVLW data do not resolve value dependence on physical properties or gender. Therefore, the currently used definition of a normal range from 3 to 8 ml/kg seems to be invalid. Our data suggest indexing EVLW to plain body height instead of weight-based methods. As we are not aware of any abnormal hemodynamic profile for brain tumor patients, we propose our findings as a close approximation to normal values. This will require further validation in critically ill patients.

